# Dynamics of Microbial Carbon Metabolism During Vegetation Restoration in Sandy Ecosystems

**DOI:** 10.3390/microorganisms14040873

**Published:** 2026-04-13

**Authors:** Jun Yang, Yifan Yue, Xiaowei Li, Ruiheng Lv

**Affiliations:** 1 College of Horticulture and Forestry, Tarim University, Alar 843300, China; 120250117@taru.edu.cn; 2 College of Forestry and Pratacuture, Ningxia University, Yinchuan 750021, China; yueyangjun913@gmail.com; 3 State Key Laboratory Breeding Base of Land Degradation and Ecological Restoration of Northwest China, Ningxia University, Yinchuan 750021, China; 4 The National and Local Joint Engineering Laboratory of High Efficiency and Superior-Quality Cultivation and Fruit Deep Processing Technology of Characteristic Fruit Trees in South Xinjiang, Tarim University, Alar 843300, China

**Keywords:** *Caragana korshinskii*, vegetation restoration, microbial carbon metabolism, functional succession, adaptive strategies

## Abstract

Understanding the succession of soil microbial carbon metabolism functions is crucial for elucidating carbon cycling mechanisms during ecosystem restoration in sandy lands. Soils were collected from *Caragana korshinskii* shrubland sites across a restoration chronosequence (0, 10, 30, 50, and 70 years) in the Mu Us Sandy Land. Biolog carbon source utilization analysis and metagenomic sequencing were employed to characterize the successional patterns of microbial carbon metabolism functions—a shift in carbon metabolism strategies from acquisition to conservation, and a transition in functional diversity from generalism to specialization. The results indicated that microbial communities exhibited two associated successional shifts in functional characteristics: carbon source utilization tended to transition from simple to complex substrates, while functional gene expression showed a progressive shift from broad multi-pathway patterns toward pathway-specific specialization. AWCD values increased continuously with restoration duration, and carbon source utilization patterns diverged significantly around 30 years. Early-stage sites (0–30 years) primarily utilized simple carbon sources, whereas late-stage sites (50–70 years) shifted toward more complex and diverse substrates. Principal component analysis revealed that 27 carbon sources contributed 91.3% of the variance to PC1. Microbial community structure succession revealed that *Actinobacteria* peaked at 10 years (43.63%), *Proteobacteria* peaked at 30 years (45.66%), and taxa such as *Bacilli* and *Solirubrobacter* dominated at 50–70 years. Carbon metabolism pathways exhibited stage-specific succession: glycolysis and the ED pathway were active in early stages, acetate metabolism dominated with the 3HB cycle peaking in intermediate stages, and the CBB cycle increased in later stages while methane metabolism shifted from high to low contribution. These two associated successional shifts occurred along the same restoration chronosequence, with the progressive transition in substrate utilization accompanying the development of specialist functional characteristics. These findings provide insights into the successional dynamics of microbial carbon metabolism during vegetation restoration, offering a microbiological basis for optimizing ecological restoration practices and enhancing soil carbon sequestration in sandy lands.

## 1. Introduction

Soil microorganisms are the core drivers of material cycling and energy flow in terrestrial ecosystems, playing an irreplaceable role in global carbon cycling processes. Soil microorganisms participate in carbon cycling through multiple pathways, including decomposing organic matter, fixing atmospheric CO_2_, and regulating soil carbon pools, with their metabolic activities directly influencing the accumulation, transformation, and distribution of soil carbon [1,2]. Different microbial taxa, such as bacteria and fungi, serve as primary drivers of soil organic matter decomposition, capable of breaking down various organic compounds ranging from simple sugars to complex lignin [3]. Studies have shown that microbial-mediated carbon transformation processes exert a decisive influence on soil carbon pool stability, with the total amount of organic carbon stored in global soils far exceeding the carbon content in the atmosphere [4]. Therefore, revealing the functional characteristics of soil microbial carbon metabolism is of great significance for understanding carbon cycling mechanisms in terrestrial ecosystems.

Sandy land ecosystems, as an important component of global terrestrial ecosystems, play a unique and critical role in carbon cycling. Global drylands cover approximately 45% of the Earth’s land area and store 646 Pg of soil organic carbon and 1237 Pg of soil inorganic carbon to 2 m depth, strongly impacting the global carbon stock and contributing to the land-based carbon sink [5]. Furthermore, dryland ecosystems are the dominant driver of inter-annual variability in the global land carbon sink, creating a negative feedback through carbon sequestration [6]. Compared with other ecosystems, sandy land ecosystems possess fragile ecological environments, sparse vegetation coverage, and extreme climatic conditions, making their carbon sequestration processes face greater challenges [7,8]. Vegetation restoration has been proven an effective approach for enhancing carbon sequestration capacity in sandy lands [9]. By increasing above- and belowground biomass, improving soil structure, and promoting soil microbial activity, vegetation restoration significantly increases carbon storage in sandy land ecosystems [10]. The Mu Us Sandy Land, as one of China’s four major sandy lands, represents a key region for desertification control in the country. During the vegetation restoration process, *Caragana korshinskii*, with its drought tolerance, barren-soil tolerance, and nitrogen-fixing capacity, has become a pioneer species for sandy land ecological restoration [11]. Research indicates that with increasing establishment duration of *C. korshinskii*, its capacity for wind erosion control and water conservation gradually strengthens [12,13], while significantly promoting increases in soil microbial diversity and abundance [14]. This provides an ideal research platform for understanding microbial functional evolution during vegetation restoration.

During ecosystem restoration processes, soil microbial communities undergo succession not only in composition and diversity but also experience profound transformations in their carbon metabolism functional characteristics. Changes in microbial community structure provide the biological foundation for functional succession [15], while shifts in functional characteristics directly determine the regulatory effects of microorganisms on soil carbon cycling. This functional succession manifests at multiple levels: microbial biomass and its stoichiometric characteristics reflect community nutrient demand and utilization efficiency [16]; changes in extracellular enzyme activities reveal microbial capacity for decomposing and transforming organic matter [17,18]; shifts in carbon source utilization patterns reflect microbial metabolic preferences for different substrates [19]; and the expression of carbon metabolism pathways and functional genes directly determines the efficiency of microbial-mediated carbon transformation [20,21]. Previous studies have shown that during forest and grassland ecosystem restoration, microbial carbon metabolism functions exhibit a transition trend from r-strategy to K-strategy [22], shifting from rapid growth and high metabolic activity characteristic of resource acquisition toward slow growth and efficient utilization characteristic of resource conservation. However, studies in sandy land ecosystems have largely focused on microbial community composition and diversity, with relatively limited attention to the successional dynamics of carbon metabolism strategies during long-term vegetation restoration. Therefore, this study examined *C. korshinskii* shrubland sites across a restoration chronosequence (0, 10, 30, 50, and 70 years) in the Mu Us Sandy Land, using Biolog carbon source utilization analysis and metagenomic sequencing to investigate the successional characteristics of soil microbial carbon metabolism functions. The aim was to reveal the transformation patterns of microbial carbon metabolism strategies, providing a scientific basis for sandy land ecological restoration.

## 2. Materials and Methods

### 2.1. Study Area

The study area is located in Yanchi County, Ningxia, on the southern edge of the Mu Us Sandy Land (37°04′–38°10′ N, 106°30′–107°41′ E), situated in the semi-arid region of China with a typical continental monsoon climate. The mean annual temperature is 8.1 °C, with annual precipitation of 280 mm, primarily concentrated from July to September, and annual evaporation of 2100 mm. The soil type is predominantly aeolian sandy soil, characterized by loose texture and low organic matter content. Large-scale vegetation restoration efforts have been implemented in this region since the 1950s, with *C. korshinskii* extensively planted as the primary sand-fixing species, forming temporal gradients of different restoration durations.

### 2.2. Site Selection and Sample Collection

Based on the planting time of *C. korshinskii* and local forestry department records, mobile sand dunes (0 year) were selected as the control, along with *C. korshinskii* sand-fixing shrublands with restoration durations of 10, 30, 50, and 70 years as study subjects. For each restoration duration, three spatially independent replicate plots (20 m × 20 m) were established, with inter-plot distances exceeding 500 m. Within each plot, five sampling points were selected using an S-shaped pattern, and the collected soil samples were thoroughly mixed to form one composite sample, yielding three composite samples per restoration duration for subsequent analyses. Sample collection was conducted in August 2023 during the peak growing season.

Within each plot, five sampling points were randomly selected using an S-shaped sampling pattern, and soil samples from the 0–20 cm layer were collected using a soil auger. The five soil samples from the same plot were thoroughly mixed, and approximately 2 kg was obtained as a composite sample using the quartering method. Upon returning to the laboratory, soil samples were cleared of plant residues and gravel, then divided into three portions: one portion was stored at 4 °C for microbial biomass and enzyme activity measurements; another portion was preserved in an ultra-low temperature freezer at −80 °C for DNA extraction and metagenomic sequencing; and the remaining samples were air-dried, ground, and sieved (2 mm and 0.15 mm) for physicochemical property measurements.

### 2.3. Experimental Methods

#### 2.3.1. Biolog Carbon Source Utilization Analysis

Biolog-EcoPlate™ microplates were employed to assess microbial carbon source utilization capacity [23]. Fresh soil samples (5 g) were weighed and combined with 45 mL sterile saline solution, shaken for 30 min, and then allowed to settle. The supernatant was diluted 100-fold, and 125 μL was inoculated into each well of the Eco plate (containing 31 carbon sources), followed by incubation at 25 °C in darkness. Absorbance values (wavelengths 590 nm and 750 nm) were measured every 24 h, with continuous monitoring for 168 h.

The average well color development (AWCD) was calculated to characterize microbial activity:

$$AWCD=\frac{\sum_{i=1}^{31}\left(c_{i}-R\right)}{31}$$

where *C_i_* represents the net absorbance of the *i*-th carbon source well and *R* represents the net absorbance of the blank control. Data from 72 h of incubation were selected for principal component analysis.

#### 2.3.2. Amplicon Sequencing

Genomic DNA was extracted from soil samples using the CTAB method, and DNA purity and concentration were verified by 1% agarose gel electrophoresis. Samples were diluted to 1 ng/µL with sterile water prior to amplification. Bacterial 16S rDNA was amplified using primers 515F (5′-GTGCCAGCMGCCGCGGTAA-3′) and 806R (5′-GGACTACHVGGGTWTCTAAT-3′), targeting the V4 hypervariable region. Fungal ITS regions were amplified using primers ITS5-1737F (5′-GGAAGTAAAAGTCGTAACAAGG-3′) and ITS2-2043R (5′-GCTGCGTTCTTCATCGATGC-3′). PCR amplification was performed in a 30 µL reaction volume containing 15 µL Phusion^®^ High-Fidelity PCR Master Mix with GC Buffer (NEB), 1 µL of each forward and reverse primer (1 µM), 10 µL template DNA (10 ng), and 2 µL sterile water. The thermal cycling conditions were as follows: initial denaturation at 98 °C for 1 min; 30 cycles of 98 °C for 10 s, 50 °C for 30 s, and 72 °C for 30 s; followed by a final extension at 72 °C for 5 min. PCR products were purified by 2% TAE agarose gel electrophoresis, and target bands were recovered using a Universal DNA Purification Kit (TianGen, Beijing, China). Equal quantities of purified PCR products were pooled and sequenced on the Illumina platform using the NEB Next^®^ Ultra DNA Library Prep Kit for paired-end 150 bp sequencing, with a minimum sequencing depth of 50,000 reads per sample. Raw sequencing data were processed through quality filtering, primer removal, paired-end merging, and chimera removal, followed by denoising using the DADA2 plugin within the QIIME2 platform (v2019.1) to generate high-quality amplicon sequence variant (ASV) tables. Bacterial ASVs were taxonomically annotated against the Greengenes database (v13.8, 99% similarity, trimmed to the V4 region using 515F/806R primers), and fungal ASVs were annotated against the UNITE database (v8.2). Sequences annotated as chloroplasts, mitochondria, or those that could not be classified at the kingdom level were removed from subsequent analyses.

#### 2.3.3. Metagenomic Sequencing and Analysis

Total microbial DNA was extracted from 0.5 g of each soil sample using a modified CTAB method [24]. DNA quality, concentration, integrity, and purity were assessed by 1% agarose gel electrophoresis and Agilent 5400. Qualified samples were used to construct libraries with the NEBNext^®^ Ultra™ DNA Library Prep Kit (Catalog #E7370L, NEB, Ipswich, MA, USA). DNA was fragmented to 350 bp using a Covaris ultrasonicator, followed by end repair, A-tailing, adapter ligation, size selection, and PCR amplification. Qualified libraries were sequenced on the Illumina platform using a paired-end 150 bp (PE150) strategy. Each sample generated an average of approximately 7.5 Gb of raw data (average 25 million raw reads per sample). After quality control, an average of 22.8 million high-quality clean reads were retained per sample (average retention rate 91%, Q30 > 98%), ensuring sufficient sequencing depth for downstream metagenomic analysis.

Raw sequencing data were quality-controlled using Kneaddata. Adapter sequences, low-quality reads (quality score ≤ 20), and sequences shorter than 50 bp were removed using Trimmomatic. Host-derived sequences were filtered using Bowtie2, and quality control efficiency was evaluated using FastQC. Taxonomic annotation was performed using Kraken2 against a custom microbial nucleotide database, with Bracken used to estimate the actual relative abundance of species in each sample. Functional annotation was performed using HUMAnN3, which aligned quality-controlled reads against the UniRef90 protein database via DIAMOND. Functional profiles and relative abundances were obtained based on the ID correspondence between UniRef90 and the KEGG database, with a focus on glycolysis (ko00010), citrate cycle (ko00020), pentose phosphate pathway (ko00030), pyruvate metabolism (ko00620), carbon fixation pathways in prokaryotes (ko00720), and methane metabolism (ko00680).

### 2.4. Data Processing and Statistical Analysis

Raw data were organized using Excel 2019 and R v4.0.3. LEfSe (Linear discriminant analysis Effect Size) was employed to identify differential microbial taxa across different vegetation restoration durations. Non-parametric Kruskal–Wallis rank-sum tests were first used to identify microbial taxa with significant differences among groups (*p* < 0.05), followed by linear discriminant analysis (LDA) to estimate the effect size of each taxon, with LDA score > 4 considered statistically significant. Principal component analysis (PCA) was performed to reveal differences in the utilization of 31 carbon sources by soil microbial communities across different restoration durations, completed using Origin 2024 software. Metagenomic sequencing data were analyzed using the Bioinfo Cloud Technology online metagenomics analysis platform (https://www.bioincloud.tech/, accessed on 13 August 2024). Gene sets were aligned to the KEGG database, with focused analysis on carbon metabolism-related pathways, including glycolysis/gluconeogenesis (ko00010), citrate cycle (ko00020), pentose phosphate pathway (ko00030), pyruvate metabolism (ko00620), carbon fixation pathways in prokaryotes (ko00720), and methane metabolism (ko00680). LEfSe analysis (LDA score ≥ 2.0) was employed to identify significantly differential genes across all five restoration stages (0, 10, 30, 50, and 70 years) simultaneously. Genes identified as biomarkers for a given restoration stage represent KO features with significantly higher relative abundance at that stage compared to all other stages. The resulting KEGG pathway maps, in which gray boxes indicate genes detected in the samples and colored boxes represent stage-specific biomarker genes, are provided in Supplementary Figures S1–S9.

## 3. Results

### 3.1. Changes in Microbial Community Diversity with Restoration Duration

**Figure 1:**
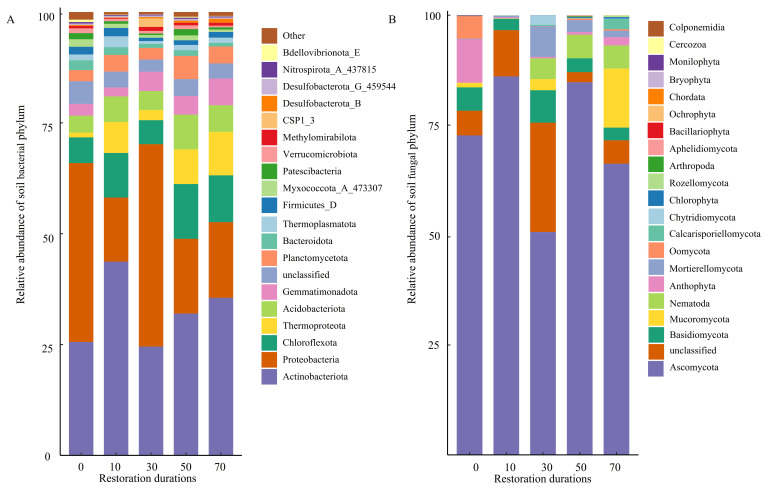
Relative abundance of soil bacterial (**A**) and fungal (**B**) phylum at different restoration durations (%).

First bullet; In the bacterial community, the dominant phyla were Actinobacteriota, which reached its maximum at 10 years of restoration (43.63%), and Proteobacteria, which peaked at 30 years of restoration (45.66%). For the fungal community, the dominant phylum was Ascomycota, reaching its maximum at 10 years of restoration (86.08%) (Figure 1).

**Figure 2:**
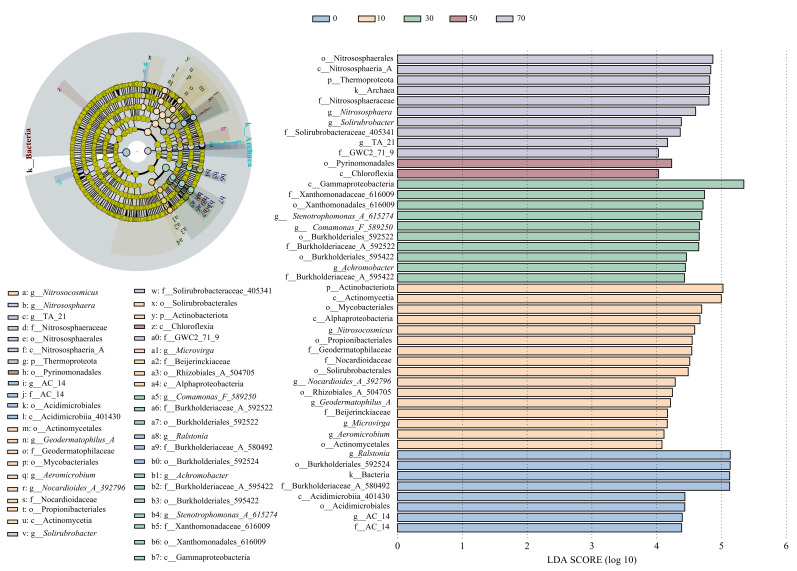
LEfSe-identified differential bacterial taxa across the restoration chronosequence.

A total of 46 significantly different bacterial taxa were identified across different restoration durations (Figure 2), with 7, 14, 7, 10, and 8 biomarkers at 0, 10, 30, 50, and 70 years, respectively. The phylogenetic tree revealed the taxonomic distribution characteristics of these differential microorganisms. At 0 years, taxa were primarily concentrated in Burkholderiales and *Ralstonia* within Proteobacteria. Biomarkers at 10 years of restoration were mainly distributed within Actinobacteriota, including Actinobacteriota and Alphaproteobacteria. Biomarkers at 30 years were predominantly concentrated in Gammaproteobacteria, such as Gammaproteobacteria and Xanthomonadaceae. At 50 years, biomarkers were distributed among taxa including Blastocatellia and Phycisphaerae, while at 70 years, they were mainly concentrated in taxa such as Solirubrobacter and Thermoleophilia.

**Figure 3:**
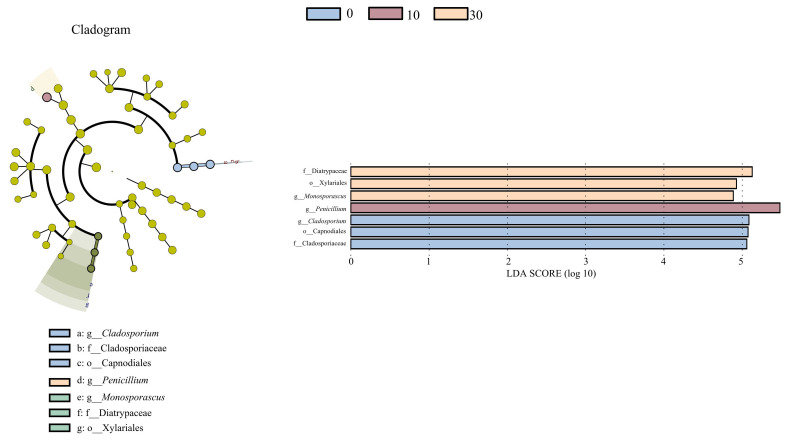
LEfSe-identified differential fungal taxa across the restoration chronosequence.

The fungal community exhibited 7 differential taxa across different restoration durations (Figure 3). Phylogenetic tree analysis indicated that biomarkers from the 0-year restoration sites, including *Cladosporium*, Cladosporiaceae, and Capnodiales, formed a closely related clade in the evolutionary tree, all belonging to Dothideomycetes within Ascomycota. The biomarker *Penicillium* at 10 years of restoration occupied a relatively independent branch. Biomarkers at 30 years, including *Monosporascus*, Diatrypaceae, and Xylariales, were distributed on another evolutionary branch, suggesting that fungal community phylogenetic structure exhibited an increasingly diverse trend with restoration durations.

### 3.2. Succession of Microbial Carbon Source Utilization Functions

**Figure 4:**
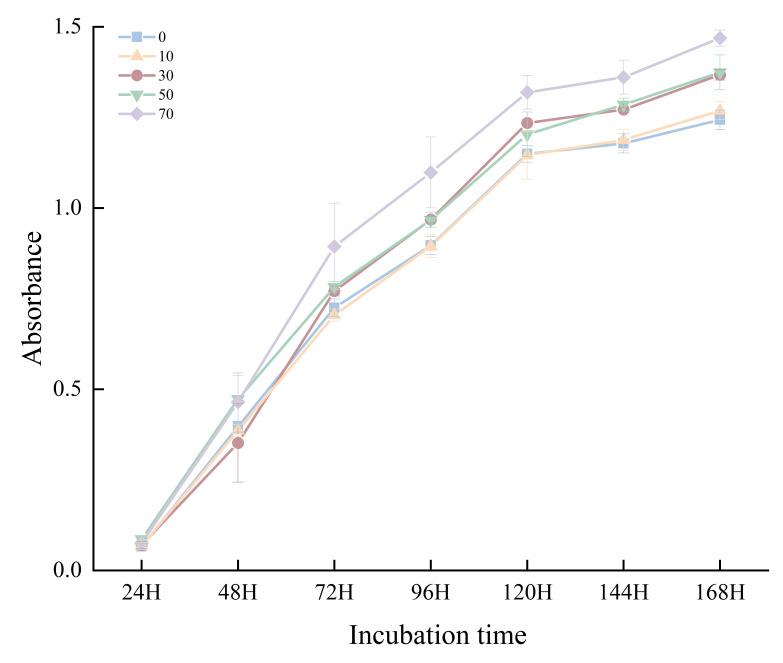
Temporal changes in AWCD of soil microbial communities during incubation.

AWCD serves as an indicator representing the utilization capacity and metabolic activity changes of soil microbial communities toward 31 carbon sources, reflecting the physiological metabolism of soil microorganisms. Higher AWCD values indicate greater microbial activity and density. As shown in Figure 4, the AWCD values of soil across different restoration durations increased with incubation time, exhibiting an S-shaped trend. All samples showed rapid increases during 24–120 h, with the maximum growth rate occurring at 72 h, followed by a decreased growth rate after 120 h. Throughout the incubation period, soil microorganisms began utilizing the 31 carbon sources after 24 h, demonstrating a lag phase.

**Figure 5:**
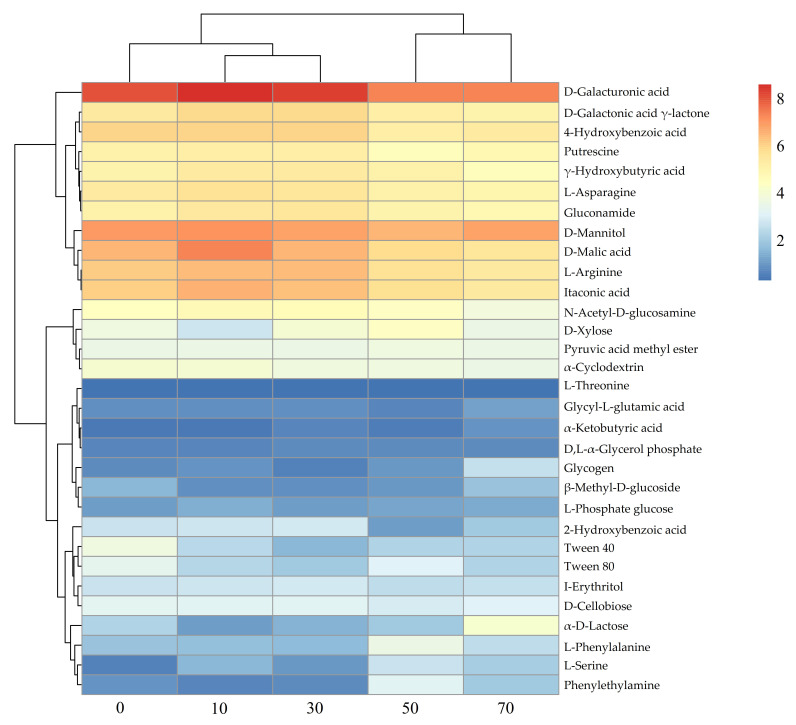
Utilization rates of different carbon sources by soil microbial communities.

Metabolic fingerprinting refers to the capacity of soil microorganisms to utilize different carbon sources on microplates. Cluster analysis was employed to reveal differences in the relative utilization intensity of various carbon sources by microbial communities in soil samples across different restoration durations (Figure 5). Soil microbial communities showed a gradual shift in carbon source utilization patterns along the restoration chronosequence, with early-stage sites (0–30 years) exhibiting higher utilization of D-galacturonic acid, D-mannitol, and D-malic acid, while late-stage sites (50–70 years) showed relatively lower utilization of D-xylose.

Based on three aspects—organic compound chemical functional groups, microbial physiological metabolic pathways, and ecological functions—the 31 carbon source substrates on the ECO plate were classified into six major categories: carbohydrates and their derivatives (12 types), carboxylic acids (5 types), amino acids (6 types), polymers (4 types), phenolic acids (2 types), and amines (2 types) (Table 1). Principal component analysis was employed to assess the metabolism of different carbon sources by microbial communities along the restoration gradient, with higher principal component loadings indicating stronger utilization of the corresponding carbon source. PC1 and PC2 accounted for 91.3% and 3.4% of the variance, respectively, totaling 94.7%. Twenty-seven carbon sources contributed substantially to PC1, including 10 carbohydrates (with D-galacturonic acid and D-mannitol as the most relevant carbon sources), 6 amino acids (with L-arginine and L-asparagine as the most relevant carbon sources), 4 carboxylic acids (with α-ketobutyric acid and D-malic acid as the most relevant carbon sources), 3 polymers (with glycogen and 2-hydroxybenzoic acid as the most relevant carbon sources), 2 phenolic acids (with 2-hydroxybenzoic acid and 4-hydroxybenzoic acid as the most relevant carbon sources), and 2 amines (with phenylethylamine and putrescine as the most relevant carbon sources). All carbon sources contributed relatively little to PC2, with 2-hydroxybenzoic acid and glucose-1-phosphate being the most relevant carbon sources.

**Table 1:** Principal component loading factors of 31 carbon sources.

Carbon Source Category	Carbon Source Type	PC1	PC2
Carbohydrates	β-Methyl-D-glucoside	−3.87	−0.47
	D-Galactonic acid γ-lactone	3.53	−0.21
	D-Xylose	0.43	0.58
	D-Galacturonic acid	7.81	0.51
	I-Erythritol	−1.43	−0.78
	D-Mannitol	5.90	0.25
	N-Acetyl-D-glucosamine	1.57	−0.46
	Gluconamide	2.98	−0.49
	D-Cellobiose	−0.66	−0.57
	L-Phosphate glucose	−3.95	−0.91
	α-D-Lactose	−2.09	0.51
	D,L-α-Glycerol phosphate	−4.84	−0.75
Amino acids	L-Arginine	4.43	−0.33
	L-Asparagine	3.19	0.10
	L-Phenylalanine	−1.97	0.90
	L-Serine	−3.18	2.26
	Glycyl-L-glutamic acid	−4.52	−0.55
	L-Threonine	−5.51	−1.14
Carboxylic acids	Pyruvic acid methyl ester	0.26	0.34
	γ-Hydroxybutyric acid	2.79	−0.02
	Itaconic acid	4.41	−0.24
	α-Ketobutyric acid	−5.02	−0.48
	D-Malic acid	4.99	−0.27
Polymers	Tween 40	−1.74	0.09
	Tween 80	−1.47	0.08
	α-Cyclodextrin	0.60	−0.42
	Glycogen	−3.93	0.81
Phenolic acids	2-Hydroxybenzoic acid	−2.00	0.93
	4-Hydroxybenzoic acid	3.90	−0.31
Amines	Phenylethylamine	−3.27	1.17
	Putrescine	2.677	−0.13

### 3.3. Formatting of Mathematical Components Stage-Specific Succession of Microbial Carbon Metabolism Pathways

**Figure 6:**
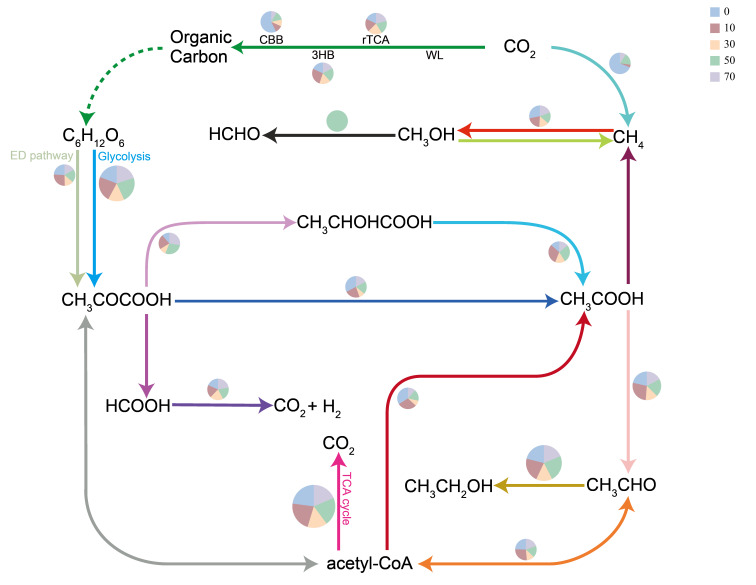
Carbon cycling network and relative contribution distribution in soils under different restoration durations. Note: The size of pie charts represents the total relative abundance of each pathway, and different colors in the pie charts represent the contribution proportion of samples from different restoration durations. CBB: Calvin-Benson-Bassham cycle; rTCA: reductive tricarboxylic acid cycle; WL: Wood-Ljungdahl pathway; 3HB: 3-hydroxybutyrate cycle.

In carbon fixation pathways, the CBB cycle exhibited higher contribution at 0 years, declined during 10–30 years, and increased again at 50 years, while the 3HB cycle peaked at 10 years. Acetyl-CoA, serving as a central metabolic intermediate, was predominantly generated through acetate metabolism at 10 years, with carbohydrates converted to pyruvate via glycolysis or the ED pathway before further transformation into acetyl-CoA. Regarding methane metabolism, CO_2_ to CH_4_ conversion showed higher contributions at 0 and 50 years, while CH_4_ to CH_3_OH conversion was active during 0–10 years. In carbohydrate metabolism pathways, both glycolysis and the ED pathway maintained relatively high contributions during 0–10 years, decreasing at 30 years, and glycolysis exhibited a slight recovery during 50–70 years (Figure 6).

Key metabolic genes exhibited distinct stage-specific differential characteristics across different restoration durations (Table 2). In the early restoration stage (0 years), the pgm gene showed significant differences across multiple pathways including glycolysis, pentose phosphate pathway, and starch and sucrose metabolism, reflecting the broad-spectrum characteristics of microbial basal metabolism. At 30 years of restoration, the aceB and E2.3.1.9 genes became signature differential genes across multiple metabolic pathways, with aceB participating in the glyoxylate cycle and pyruvate metabolism, and E2.3.1.9 involved in acetyl-CoA transformation, indicating that metabolic functions began to differentiate toward organic acid metabolism. The characteristic differential genes at 50 years of restoration were GPI and accE, encoding glucose-6-phosphate isomerase and pyruvate dehydrogenase complex respectively, reflecting the refined regulation of carbohydrate metabolism. At 70 years of restoration, the porA gene exhibited significant differences across multiple pathways including glycolysis, citrate cycle, and carbon fixation. This gene encodes pyruvate ferredoxin oxidoreductase, playing a pivotal role in connecting different metabolic pathways and reflecting the high integration of the microbial carbon metabolism network.

## 4. Discussion

### 4.1. Stage-Dependent Shifts in Microbial Community Composition and Carbon Source Utilization Along the Restoration Chronosequence

In the early restoration stage (0–30 years), SOC content was only 0.36–5.74 g·kg^−1^ and STN content was only 0.04–0.12 g·kg^−1^ (Table S1), indicating that soil resources were limited at this stage. Against this background, LEfSe analysis identified *Ralstonia* as a key indicator taxon at 0 years; this bacterium is known for its strong adaptability to disturbed and resource-poor environments [24]. The fungal community at 0 years was characterized by *Cladosporium*, which similarly demonstrates strong adaptability to barren and arid conditions [25]. Actinobacteriota reached its maximum relative abundance at 10 years (43.63%), with taxa such as *Aeromicrobium* serving as indicator organism, and are known for their capacity to decompose complex organic matter [26,27]. For 30 years, the bacterial community has been mainly characterized by the γ-Proteobacteria class. These γ-Proteobacteria groups play a significant role in vegetation restoration. They have the ability to degrade various organic pollutants such as polycyclic aromatic hydrocarbons and phenolic compounds, and play a key role in promoting the accumulation and transformation of organic matter in sandy soil [28], while the fungal community was characterized by *Monosporascus*, Diatrypaceae, and Xylariales, taxa known for their ability to decompose recalcitrant organic matter such as lignin [29]. Carbon source utilization analysis further indicated that sites at 0–30 years primarily exhibited higher utilization of simple, easily degradable carbon sources such as D-galacturonic acid, D-mannitol, and D-malic acid, while showing relatively lower utilization of more complex carbon sources such as L-serine, phenylethylamine, and α-D-lactose. Taken together, the community composition, soil physicochemical properties, and carbon source utilization data suggest that, under conditions of extreme soil resource limitation in the early restoration stage, microbial communities tended to preferentially utilize simple, readily available carbon sources.

In the late restoration stage (50–70 years), SOC content increased significantly to 7.08–8.87 g·kg^−1^ and DOC content reached 159.20–183.23 mg·kg^−1^ (Table S1), indicating substantial accumulation of soil organic matter. As vegetation restoration progressed, the composition of soil organic matter gradually shifted from simple, easily degradable compounds toward more complex, recalcitrant forms, Planctomycetes emerged as a key indicator taxon, with its genomes known to harbor diverse carbohydrate-active enzymes associated with complex polysaccharide decomposition [30], indirectly suggesting a tendency toward utilization of more complex carbon sources at this stage. Carbon source utilization data further indicated that the utilization of relatively complex carbon sources, including L-serine, phenylethylamine, and α-D-lactose, showed a notable increase at 50–70 years. Taken together, the soil physicochemical properties, community composition, and carbon source utilization data suggest that, as vegetation restoration progressed and soil organic matter continued to accumulate, the composition of available substrates became increasingly complex, and the capacity of microbial communities to utilize complex carbon sources gradually increased accordingly, reflecting a progressive shift in substrate utilization characteristics along the restoration chronosequence. Waldrop and Firestone [31] demonstrated that the selective utilization of specific carbon sources by different microbial functional groups is closely related to their phylogenetic background and metabolic capabilities, providing theoretical support for the association between shifts in dominant taxa and changes in carbon source utilization patterns observed in this study.

### 4.2. Stage-Specific Succession of Carbon Metabolism Pathways and Functional Gene Expression

During vegetation restoration in the Mu Us Sandy Land, microbial carbon metabolism pathways exhibited distinct stage-specific characteristics, progressing from an early period of diverse metabolic activity (high proportions of glycolysis, ED pathway, and methane oxidation), through an intermediate metabolic reorganization period (acetate metabolism dominated, with the 3HB cycle peaking), to a late functional specialization period (CBB cycle recovery and partial restoration of glycolysis). The contribution ratios of the acetyl-CoA-centered metabolic network changed significantly across different restoration stages. Particularly at 10 years of restoration, acetate as a key metabolic intermediate occupied a dominant position, which may be related to the higher biomass of *C. korshinskii* at this stage, with abundant root exudates and litter providing rich acetate precursors [32,33]. The pathway from carbon dioxide to methane showed a higher contribution ratio at 0 years but decreased significantly at 10 years. The declining contribution of methane metabolism pathways may reflect the transition of microbial communities from anaerobic to aerobic metabolism, which has positive implications for reducing greenhouse gas emissions [34]. The temporal variation characteristics of carbohydrate metabolism pathways reflected microbial utilization strategies for carbon sources of different complexity. Both glycolysis and the ED pathway showed high contribution ratios at 0 and 10 years, consistent with the predominance of easily utilizable carbon sources at this stage. With increasing restoration duration, the contribution ratios of both pathways decreased, which may be related to the shift of microbial communities toward utilizing more complex carbon sources. Notably, the contribution ratio of glycolysis showed a slight recovery at 50 and 70 years, suggesting that microbial communities may have optimized metabolic pathways with higher energy efficiency [35]. This stage-specific succession pattern of microbial carbon metabolism pathways suggests a dynamic response relationship between microbial community functions and vegetation restoration processes, providing microbiological insights for deeper understanding of carbon cycling mechanisms during ecological restoration of desertified lands and for optimizing restoration strategies.

At 0 years, the most prominent feature was the differential expression of the pgm (phosphoglucomutase) gene across multiple pathways including glycolysis, pentose phosphate pathway, and starch and sucrose metabolism, reflecting a microbial “generalist” strategy to maintain maximum metabolic flexibility for rapid utilization of limited and simple carbon sources. The differential expression of genes in the citrate cycle, including IDH1 (isocitrate dehydrogenase), frdA (fumarate reductase), and sdhA (succinate dehydrogenase), suggested that microbial communities may have adjusted metabolic pathways to achieve efficient energy acquisition, a metabolic characteristic enabling microorganisms to efficiently utilize limited carbon resources [22]. At 30 years of restoration, the differential expression of the aceB (malate synthase) gene across multiple pathways, along with the activity of E2.3.1.9 (acetyl-CoA C-acetyltransferase), reflected that microbial communities began developing more diversified carbon transformation pathways. At 50 years, the differential expression of carbohydrate metabolism-related genes such as GPI (glucose-6-phosphate isomerase) and glk (glucokinase), as well as genes like aceE (pyruvate dehydrogenase E1 component),suggested a tendency toward more refined resource allocation in microbial communities and began investing in metabolic pathways with higher energy efficiency. This differentiation and complexification of metabolic pathways aligns with the findings of Zhou et al. [36] from a study of 85 global secondary successions, suggesting a progressive shift of microbial communities from “generalists” toward “specialists”. The differential expression of genes such as OGDH (alpha-ketoglutarate dehydrogenase) and leuA (alpha-isopropylmalate synthase) contributed to enhancing the metabolic efficiency and material transformation capacity of soil microbial communities by regulating carbon metabolism pathways and amino acid synthesis [37]. At 70 years of vegetation restoration, characteristics of metabolic integration emerged, with genes such as porA (pyruvate ferredoxin oxidoreductase) and ACO (aconitase) showing significant differences across pathways including the citrate cycle and carbon fixation pathways in prokaryotes. Particularly, porA, serving as a metabolic hub connecting glycolysis and the citrate cycle, showed consistent differential expression across multiple pathways, suggesting the formation of a more integrated carbon metabolism network in microbial communities. In resource limited environments, microorganisms need to employ specialized metabolic strategies to compete for limited resources, including producing extracellular enzymes capable of degrading complex organic compounds and efficiently utilizing organic matter [38].

## 5. Conclusions

This study documented two associated successional shifts in soil microbial carbon metabolism functions along a *Caragana korshinskii* restoration chronosequence: a transition in carbon source utilization from simple to complex substrates, and a progressive shift in functional gene expression from broad multi-pathway patterns toward pathway-specific specialization. Together, these observations suggest successional dynamics in microbial metabolic characteristics during vegetation restoration in sandy land. It should be noted that Biolog-EcoPlate measures potential carbon source utilization capacity under resource-sufficient conditions in vitro, and the functional gene profiles reflect potential rather than realized metabolic activity. Future studies incorporating direct measurements of microbial growth rates, metabolic quotients, and in situ carbon flux would allow more rigorous characterization of the realized metabolic strategies of microbial communities in sandy land ecosystems. Additionally, the mechanisms linking microbial community succession to soil organic carbon dynamics warrant further investigation. These findings provide insights into the functional succession of microbial carbon metabolism during vegetation restoration, offering a microbiological basis for optimizing ecological restoration practices in sandy lands.

## Figures and Tables

**Table 2:** Differential metabolic genes across carbon metabolism pathways during vegetation restoration.

Pathway	Restoration Duration			
	0	30	50	70
Glycolysis	*pgm*		*gllk*, GPI, aceE	*porA*
Citrate cycle	*frdA*, *sdhA*, IDH1	IDH3	OGDH, *aceE*	*porA*, E4.2.1.A, ACO
Pentose phosphate pathway	E2.2.1.2, *pgm*		GPI, *deoB*	
Starch and sucrose metabolism	*pgm*		GPI, *gllk*	
Propanoate metabolism	*pta*	*aceB*, E2.3.1.9	*aceE*, *leuA*	*porA*, E4.2.1.A
Glyoxylate and dicarboxylate metabolism	*purU*	E2.3.1.9, *aceB*, E4.1.3.1	ACO, *ttuC*, *gyaR*	
Pyruvate metabolism	*pta*	E2.3.1.19, ACADM	*mmsA*	*porA*
Methane metabolism	E2.3.1.8	*fdhA*, *cofH*, *cofG*		*porA*
Carbon fixation pathways in prokaryotes	E2.3.1.8, IDH, *frdA*, *sdhA*	*ccsA*, E2.3.1.9		*porA*, ACO, E4.2.1.2A

## Data Availability

The original contributions presented in this study are included in the article/Supplementary Material. Further inquiries can be directed to the corresponding authors.

## Supplementary Materials

The following supporting information can be downloaded at: https://www.mdpi.com/article/10.3390/microorganisms14040873/s1, Figure S1: Glycolysis pathway map based on KEGG database; Figure S2: Citrate cycle pathway based on KEGG database; Figure S3: Pentose phosphate pathway based on KEGG database; Figure S4: Starch and sucrose metabolism pathway based on KEGG database; Figure S5: Pyruvate metabolism pathway based on KEGG database; Figure S6: Glyoxylate and dicarboxylate metabolism pathway based on KEGG database; Figure S7: Propanoate metabolism pathway based on KEGG database; Figure S8: Methane metabolism pathway based on KEGG database; Figure S9: Carbon fixation pathways in prokaryotes based on KEGG database; Table S1: Changes in the soil abiotic and biotic factors across the desert restoration chronosequence in the Mu Us Sandy Land.
