# Supplementary material for: Dynamics of Microbial Carbon Metabolism During Vegetation Restoration in Sandy Ecosystems
Source: Microorganisms. 2026 Apr 13;14(4):873. doi: 10.3390/microorganisms14040873 (PMC13119381; doi:10.3390/microorganisms14040873)
Supplement: Supplementary file 1 [file microorganisms-14-00873-s001.zip › microorganisms-4235143-supplementary.pdf]

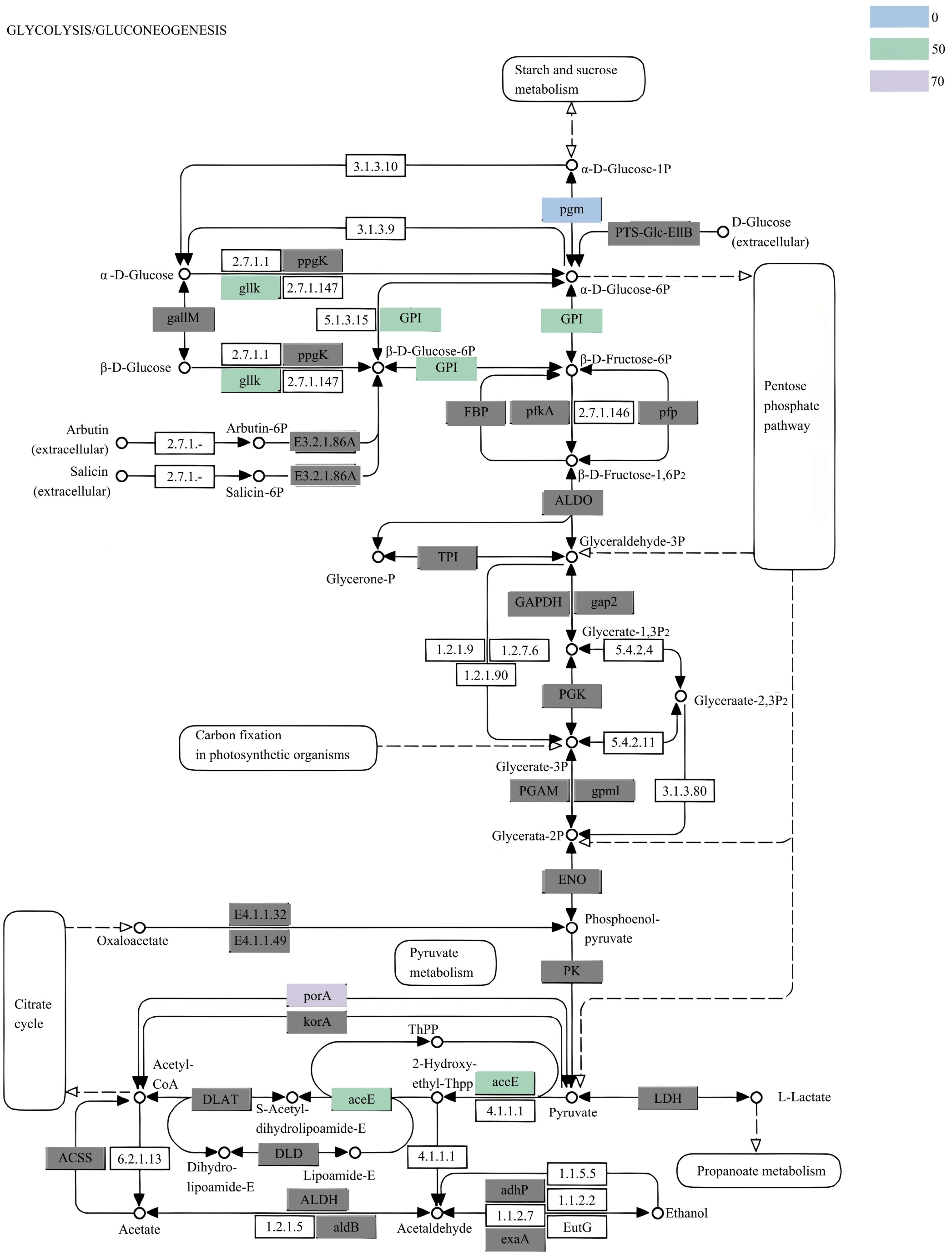

Figure S1. Glycolysis pathway map based on KEGG database

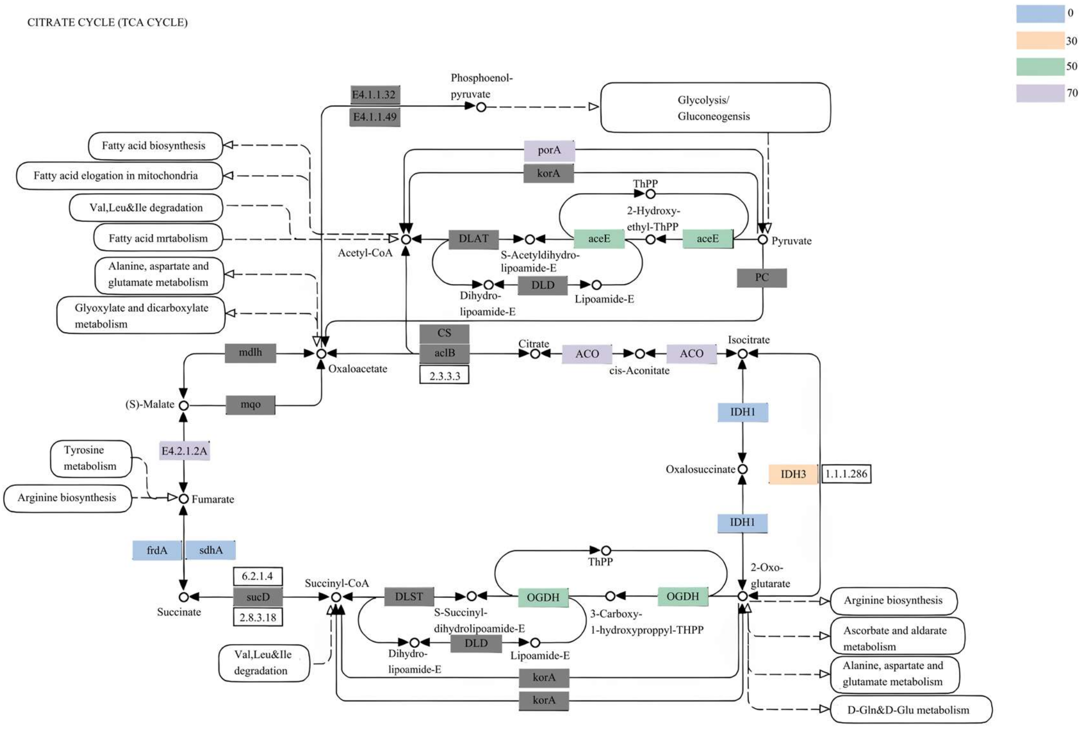

Figure S2. Citrate cycle pathway based on KEGG database

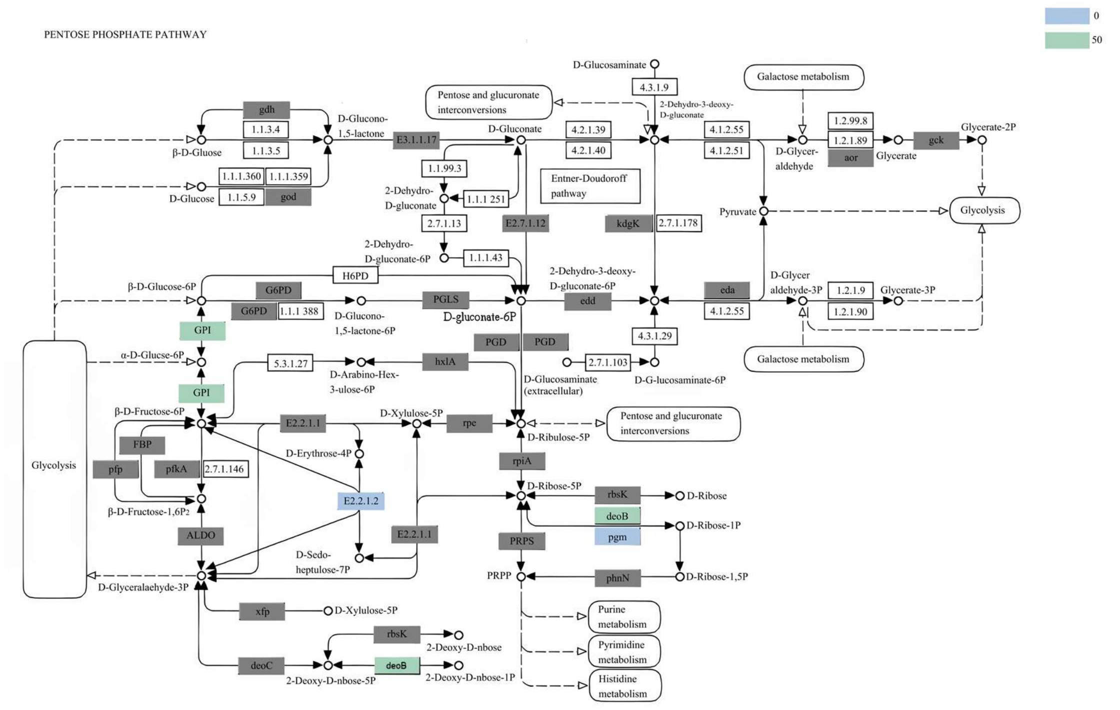

Figure S3. Pentose phosphate pathway based on KEGG database

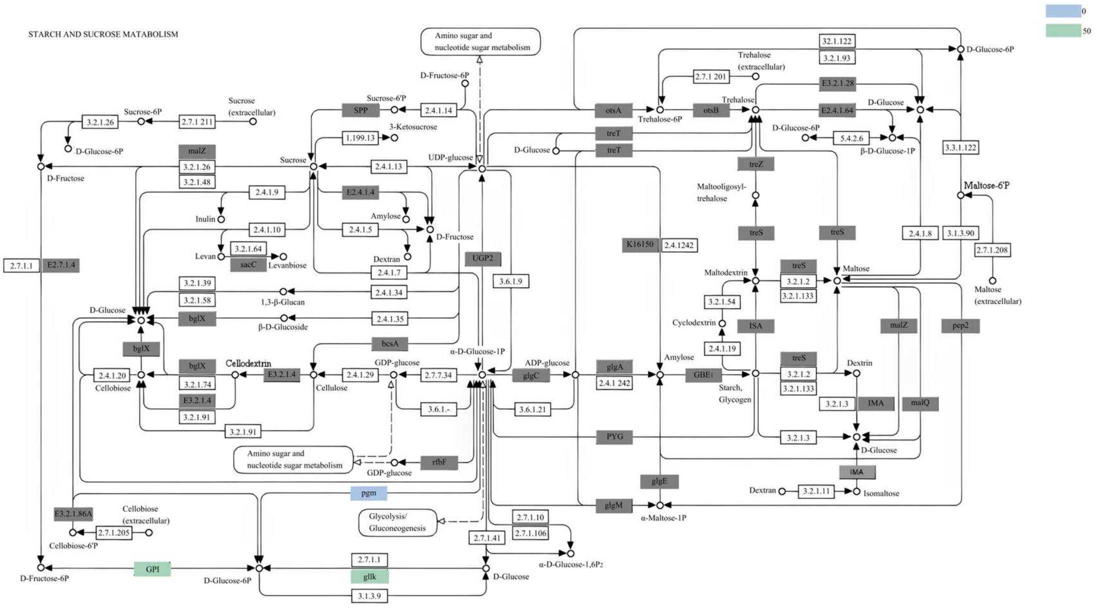

Figure S4. Starch and sucrose metabolism pathway based on KEGG database

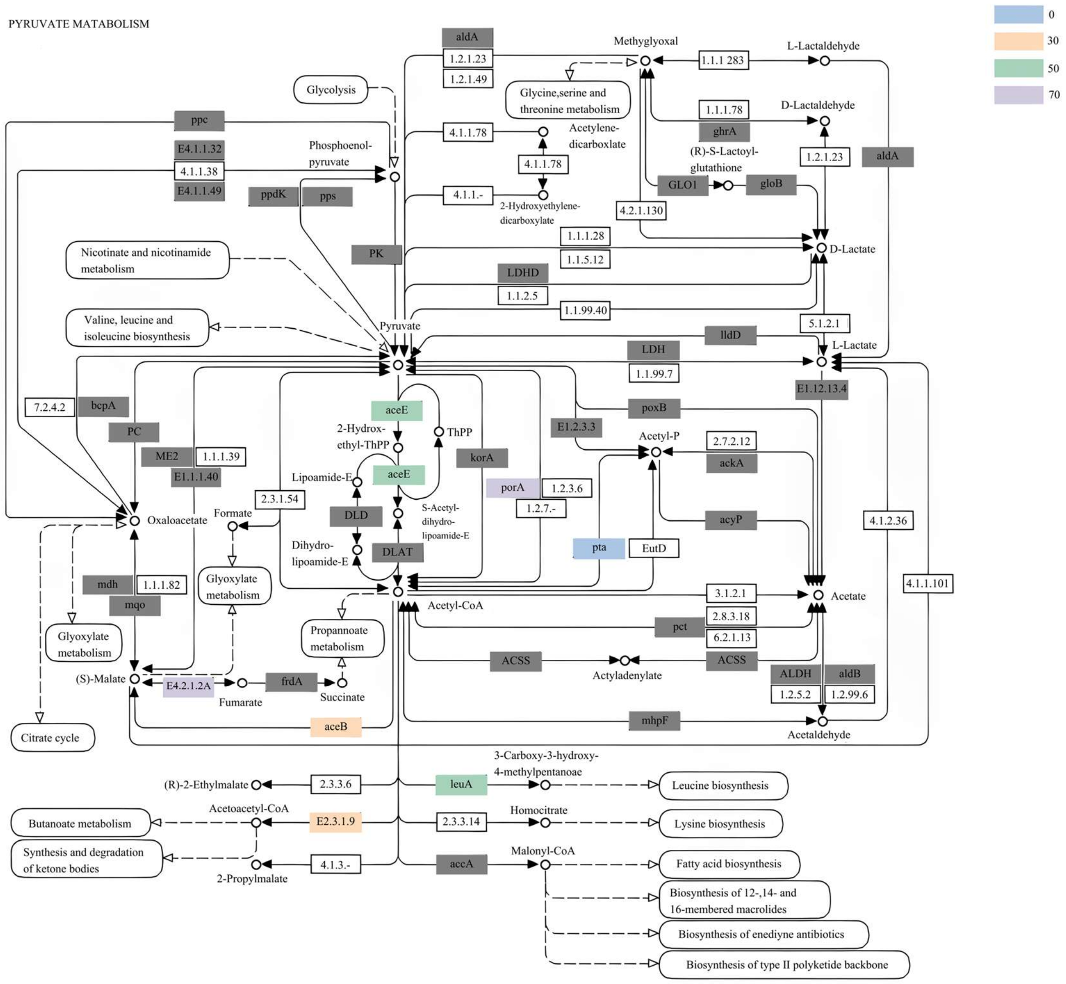

Figure S5. Pyruvate metabolism pathway based on KEGG database

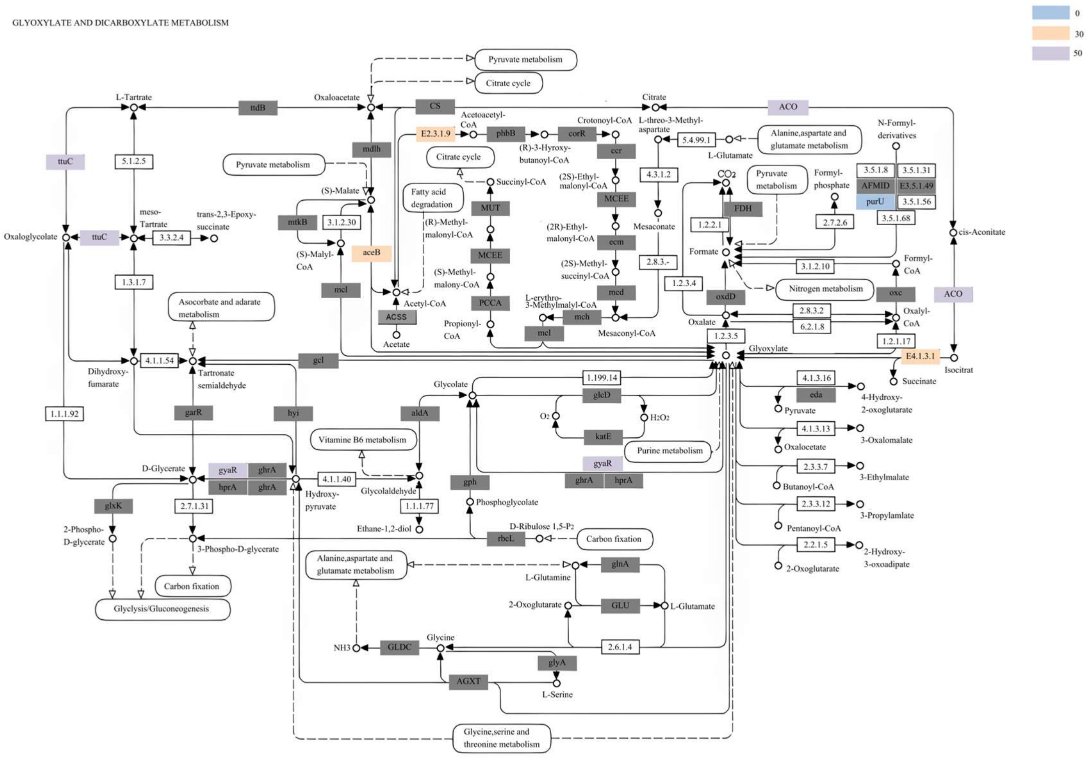

Figure S6. Glyoxylate and dicarboxylate metabolism pathway based on KEGG database

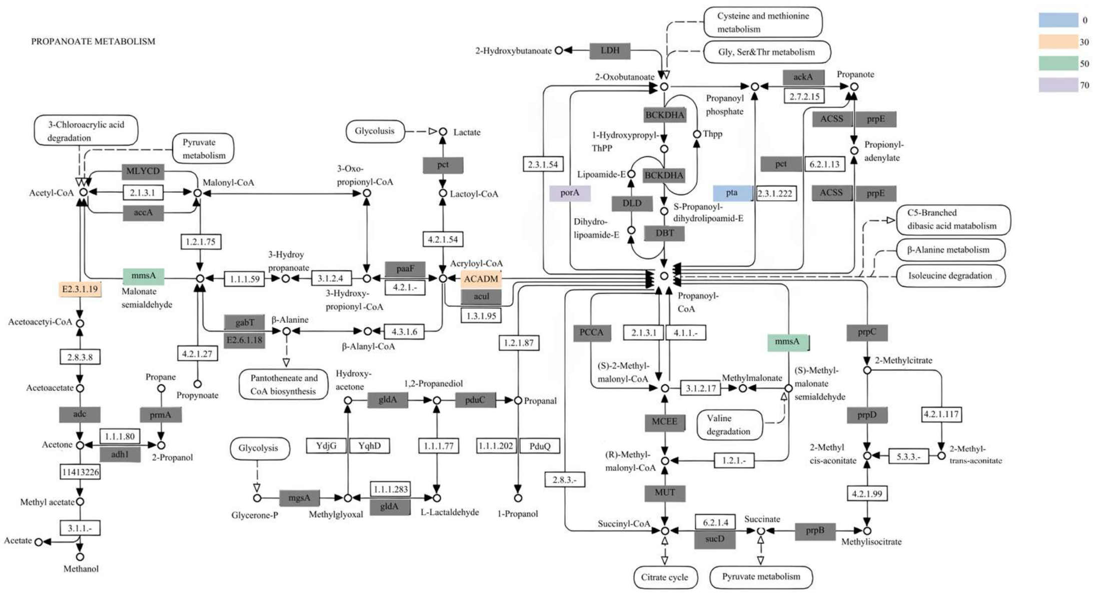

Figure S7. Propanoate metabolism pathway based on KEGG database

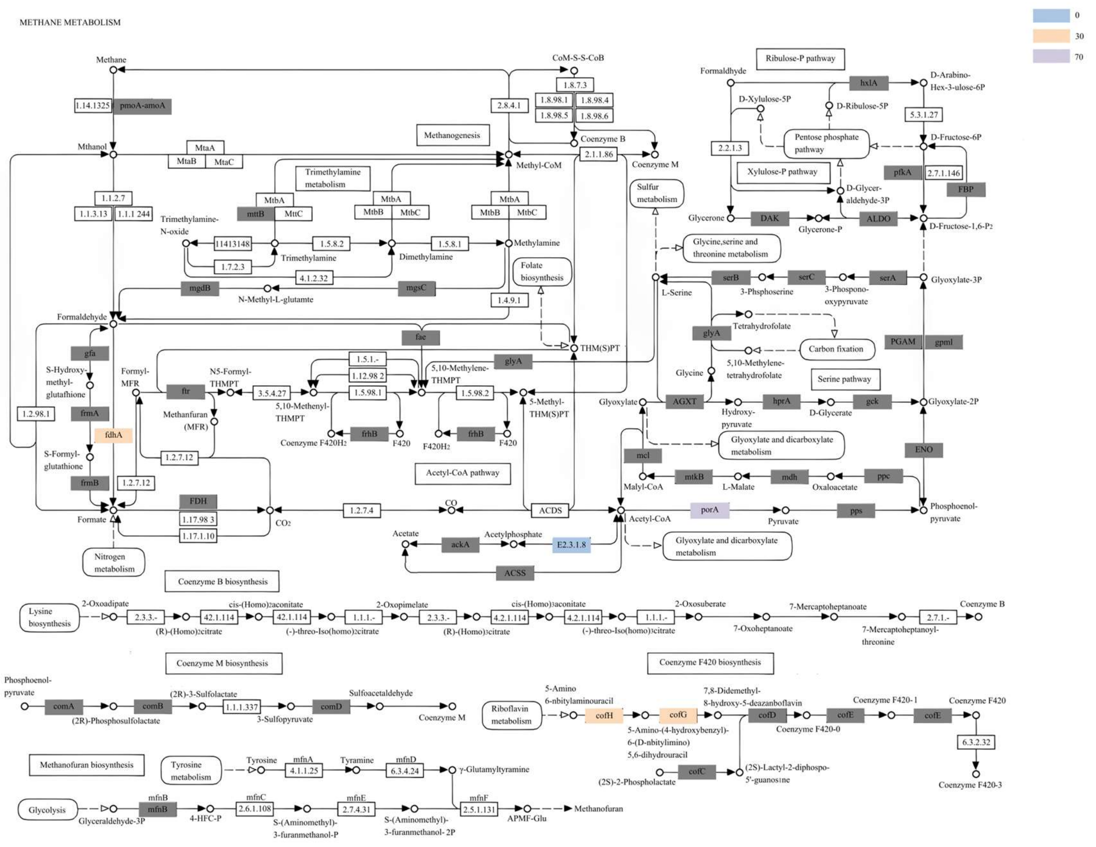

Figure S8. Methane metabolism pathway based on KEGG database

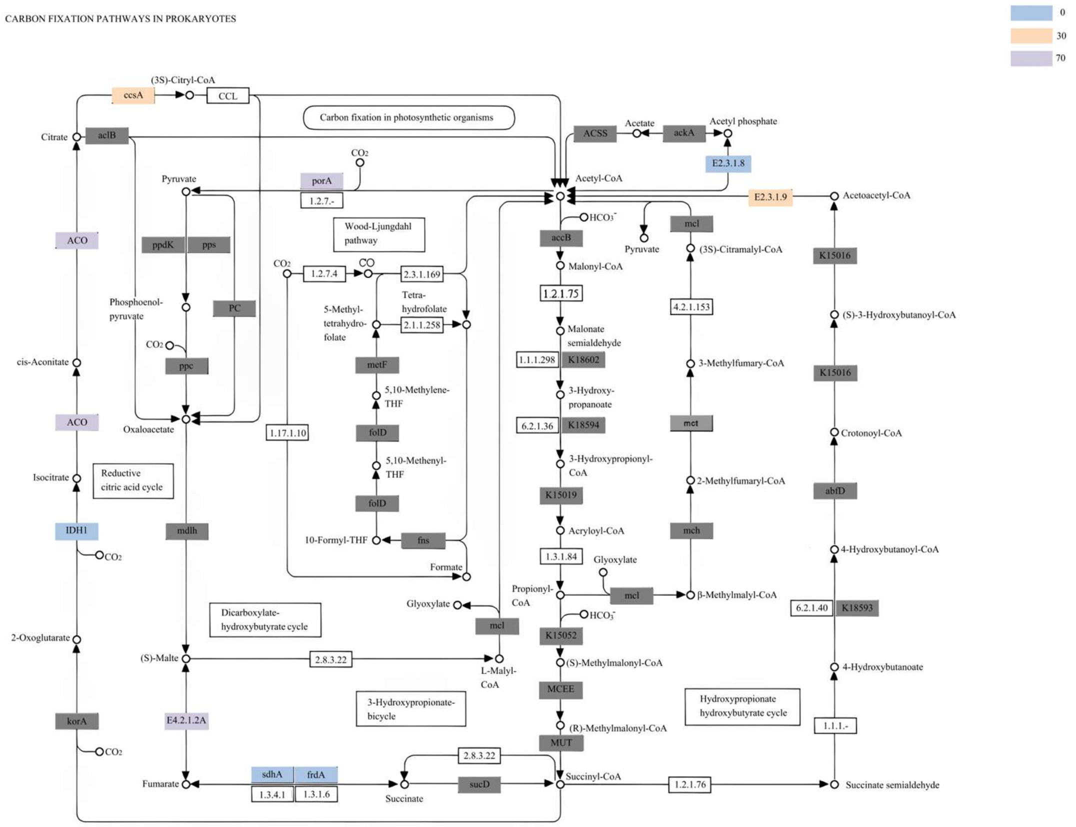

Figure S9. Carbon fixation pathways in prokaryotes based on KEGG database

Table S1. Changes in the soil abiotic and biotic factors across the desert restoration chronosequence in the Mu Us Sandy Land.

Soil factor	Years of restoration				
	0	10	30	50	70
pH	8.07±0.02d	8.26±0.07c	8.74±0.04a	8.72±0.06a	8.61±0.09b
EC ($\mu s \cdot cm^{-1}$)	27.33±5.22d	42.63±5.41c	59.46±2.51a	73.23±8.45ab	71.46±2.90b
SOC ($g \cdot kg^{-1}$)	0.36±0.10c	1.61±0.47c	5.74±0.98b	8.87±0.34a	7.08±1.49ab
DOC ($mg \cdot kg^{-1}$)	10.44±1.06b	53.93±31.23a	141.56±55.34ab	183.23±81.56a	159.20±3.02ab
STN ($g \cdot kg^{-1}$)	0.04±0.02b	0.05±0.01b	0.12±0.01a	0.11±0.01a	0.10±0.02a
STP ($g \cdot kg^{-1}$)	0.19±0.01ab	0.20±0.02a	0.19±0.01a	0.18±0.01ab	0.17±0.01b
SAP ($mg \cdot kg^{-1}$)	2.60±0.53a	2.53±0.43a	2.773±0.09a	3.33±0.55a	3.41±0.57a
AK ($mg \cdot kg^{-1}$)	168.39±23.71b	220.51±51.66b	331.14±88.60b	539.80±219.06a	279.24±11.68b

Notes: All values are the mean (± standard error). Different lowercase letters indicate a significant difference ($P < 0.05$) according to Tukey's test.

EC, soil electric conductivity; SOC, soil organic carbon; DOC, soil dissolved organic carbon; STN, soil total nitrogen; STP, soil total phosphorus; SAP, soil available phosphorus; SAK, soil available potassium.
